# School and classroom effects on Daily Physical Activity (DPA) policy implementation fidelity in Ontario classrooms: a multi-level analysis

**DOI:** 10.1186/s12889-018-5720-2

**Published:** 2018-06-27

**Authors:** Kenneth R. Allison, Anne N. Philipneri, Karen Vu-Nguyen, Heather E. Manson, John J. M. Dwyer, Erin Hobin, Bessie Ng, Ye Li

**Affiliations:** 1 KR Allison Research Consulting, 575 Windermere Avenue, Toronto, ON M6S 3L9 Canada; 2 Public Health Ontario, 480 University Avenue, Suite 300, Toronto, ON M5G 1V2 Canada; 3 The Regional Municipality of York, 50 High Tech Road, Richmond Hill, ON L4B 4N7 Canada; 4 Department of Family Relations and Applied Nutrition, University of Guelph, Macdonald Institute Building, 50 Stone Road East, Guelph, ON N1G 2W1 Canada; 5 Peel Public Health, 7120 Hurontario Street, Mississauga, ON L5W 1N4 Canada

**Keywords:** Multi-level analysis, School and classroom, Daily physical activity policy

## Abstract

**Background:**

This paper examines school and classroom effects on Daily Physical Activity (DPA) policy implementation in classrooms in Ontario, Canada. In 2005 the Ontario Ministry of Education mandated a policy requiring school boards to “ensure that all elementary students, including students with special needs, have a minimum of twenty minutes of sustained MVPA each school day during instructional time”. Based on an adaptation of Chaudoir’s conceptual framework, this paper contributes to understanding the extent to which school factors (as reported by administrators) and classroom factors (as reported by teachers) are associated with policy implementation fidelity at the classroom level.

**Methods:**

Cross-sectional online surveys were conducted in 2014 with elementary school administrators and teachers, based on representative random samples of schools and classrooms. A measure assessing implementation fidelity was developed from the six required components of the policy and for this paper fidelity at the classroom level is treated as the outcome variable. Several school- and classroom-level measures were also included in the surveys and a number of these were selected for inclusion here. Data from the two surveys were merged and selected variables were included in the multi-level analysis. Two-level logistic regression models were conducted to account for nesting of classrooms within schools and a series of models were conducted to identify factors associated with implementation fidelity.

**Results:**

The analytic sample for this study included 170 school administrators and 307 classroom teachers from corresponding schools. Findings from the multi-level logistic regression analyses indicated that only classroom/teacher-level factors were significantly associated with implementation fidelity at the classroom level. None of the school/administrator predictors were significantly related to fidelity. The most parsimonious model included five significant classroom/teacher predictors: teachers’ perception of DPA as realistic and achievable; confidence (self-efficacy); scheduling DPA in timetables; lack of space; and lack of time.

**Conclusions:**

Findings from the study indicate the theoretical and practical importance of addressing classroom and teacher factors since they are most proximal to implementation fidelity to the policy. Several of these factors also reflect complex structural and organizational contexts, indicating that a systems approach to understanding and supporting DPA implementation fidelity is warranted.

## Background

Regular physical activity (PA) participation has been shown to be beneficial for the physical and mental health of children and youth [1–4] and additionally is associated with academic achievement and increased concentration in school [5–7]. These benefits indicate the importance of participating in sufficient PA to meet international guidelines for children and youth specifying 60 min of moderate to vigorous PA (MVPA) per day [8, 9]. Yet engaging in PA on a regular basis is challenging for many children and youth and there are documented declines in PA with increasing age and among females [10, 11].

Since children and youth spend a considerable amount of time in school over several years, this is an important setting in which to provide structured opportunities for PA [4, 12, 13]. By specifying the type and amount of PA offered, school-based policies, curricula and related programs can potentially serve to level the playing field for student PA opportunities. Thus, to the degree that these initiatives are adopted and implemented, schools can provide students with an important contribution to meeting the 60 min of MVPA specified in the guidelines [14].

In order to provide school-based opportunities for children and youth to engage in PA, a number of Canadian provinces have adopted Daily Physical Activity (DPA) policies [15]. These initiatives are normally intended to augment physical and health education classes which are not, for the most part, offered each school day. In 2005 the Ontario Ministry of Education (EDU) released Policy/Program Memorandum (PPM) No. 138: Daily Physical Activity (DPA). This policy requires publicly-funded school boards to “ensure that all elementary students (grades 1-8), including students with special needs, have a minimum of twenty minutes of sustained MVPA each school day during instructional time” [16]. Unique to Ontario is the requirement that DPA be provided during instructional time – not during recess, lunch hour or after school. This provision can be seen as strengthening the initiative since it is incorporated into the curriculum as well as being a provincial policy [17].

A 2012 joint report by Cancer Care Ontario and Public Health Ontario recommended that Ontario’s DPA policy be evaluated since, although the policy was originally released in 2005, no Ontario-wide evaluation had previously been conducted and also to enhance provincial government accountability for this ongoing initiative [18]. In response to this recommendation, a research team conducted a number of sequential studies evaluating the development and implementation of DPA in Ontario [17, 19]. Evaluation of policy implementation is considered to be highly important, since implementation is conceptualized in the broader literature as a central determinant of subsequent intervention impacts and outcomes [20–22]. Implementation fidelity is described as “the degree to which an intervention was implemented as it was prescribed in the original protocol or as intended by the program developers” [23]. In the context of the current paper, fidelity refers to the degree to which DPA was implemented as specified in the policy requirements.

Consistent with Social Ecological Theory, [24] a number of factors have been previously described as being associated with implementation of school-based PA policy and curriculum initiatives. For example, intrapersonal factors, particularly at the teacher level, include relevant education and experience with PA, beliefs about the initiative’s importance, and self-efficacy/confidence [25–29]. Interpersonal factors associated with implementation include the degree to which the initiative is supported and prioritized within schools and by other organizations and individuals, such as parents [12, 25, 30, 31]. Organizational-level factors influencing implementation include training, provision of resources, access to a physical education specialist, available time, space and equipment, and lack of accountability such as monitoring [25–27, 30, 32–36]. Also, a systematic review of randomized controlled trials examining factors influencing implementation of PA interventions found time, availability of resources, school support, training, self-efficacy, teacher characteristics, and scheduling to be prominent barriers and/or facilitating factors [37].

Findings from a recent study, based on representative random sample surveys of Ontario elementary schools and classrooms, indicated that 61% of schools were implementing DPA in fidelity with the required policy, while 50% of classrooms were implementing DPA in fidelity with the requirements [17]. Additionally, survey findings (analyzed separately for schools and classrooms) indicated that several factors were significantly associated with implementation fidelity at both the school and classroom levels: awareness of policy requirements; scheduling; monitoring; use of resources and supports; perceptions that the policy is realistic and achievable; and a number of specific barriers to implementation [17]. Other factors were significantly associated with implementation fidelity at the classroom level but not the school level, such as self-efficacy for planning and implementing DPA (significant for teachers but not administrators) [17].

Descriptive findings from these surveys provided important policy-relevant information on several salient factors associated with school-level and classroom-level implementation fidelity. However, the findings from analyses of the separate surveys did not address the question of the extent to which school-level (as reported by administrators) and classroom-level (as reported by teachers) are associated with *classroom-level* DPA policy implementation fidelity. That is, what school-level factors and classroom-level factors are associated with classroom-level fidelity when controlling for their simultaneous effects? Related to this is the question of what is the most parsimonious model predicting classroom-level fidelity? These questions are highly important on both theoretical and applied levels. Conceptual models examining the various factors influencing implementation fidelity implicitly pose questions of the extent to which structural, organizational and individual factors influence implementation outcomes such as fidelity [21].

The current paper addresses these questions using multi-level analysis (MLA) of merged data from the school and classroom surveys. In relation to previous school-based studies of PA and physical education implementation, MLA has been used almost entirely in examining school and classroom influences on student self-reported PA behaviour as an outcome [31, 38]. The closest study to the current one, in both content and methods, is a report of the factors influencing classroom implementation of 15 min of PA each day in elementary schools in British Columbia (Canada) as part of the Action Schools!BC comprehensive school health initiative [25]. In that study, MLA of characteristics of schools and teachers, based on data derived from online surveys of school principals and classroom teachers, indicated that four factors were associated with implementation: level of institutionalization; receiving training; teacher self-efficacy; and attributes of the innovations [25]. Beyond this example, however, there is a gap in understanding the multi-level effects of school and classroom influences on classroom-level PA (including DPA) policy implementation. Our study contributes to a further understanding of these important questions.

In addition to their theoretical importance, these issues are also highly relevant to government and other organizations responsible for providing evidence-informed policy and program interventions/initiatives. For example, should government approaches focus more on initiatives to support and improve implementation fidelity at the school level or the classroom level? By extension, should these approaches be directed to school administrators or classroom teachers? The study reported here addresses these issues by examining both school and classroom influences on classroom-level DPA policy implementation fidelity.

## Methods

### Study design & sample

As mentioned earlier, a cross-sectional study of Ontario’s elementary schools was conducted between February and June 2014. Participants included school administrators (principals and vice principals) and grade 3, 5, and 7 teachers. These specific grades were selected in order to represent primary, junior and intermediate grade classrooms and to decrease the schools’ administrative burden of including more grades. The study used proportionate stratified random sampling to obtain a representative sample of Ontario’s publicly-funded elementary schools, based on four attributes: school board language (French versus English); school board type (public versus Catholic); location (urban versus rural), and enrolment size (≤200, 201–400, and ≥ 401).

Participant recruitment occurred in multiple stages. First, 40 school boards were approached to conduct the study in randomly sampled schools within their board and, of those, 30 agreed to participate (75% approval rate). Second, 43% of the randomly sampled schools within the respective school boards agreed to participate in the study (228 of 532 schools). An online survey was sent to one administrator from each of the 228 schools and, of those, 209 responded to the survey, resulting in a response rate of 39% (209 of 532) at the administrator level. Afterward, a random sample of 508 teachers from grade 3, 5, and 7 classrooms in schools where a school administrator responded to the survey were invited to participate in an online survey. This resulted in a 60% response rate at the teacher level (307 of 508). Additional details regarding the study methods are provided in a previous paper [17]. Ethical approval for the study was obtained from Public Health Ontario’s Ethics Review Board (ID: 2013–039.01).

### Measures

Survey measures for administrators and teachers were developed based on our adapted Chaudoir framework, [17, 21] as well as findings from interviews with key informants who were involved in the initial development and implementation of the DPA policy, [19] existing survey instruments, [39] and DPA materials published by the EDU [16, 40–43]. The survey measures and process were pre-tested and revised accordingly. The pre-test, which assessed question comprehension, skip patterns, flow and completion time, was completed by the members of the study team and the study Advisory Committee, which included representation from government, education, and public health.

The content of both administrator and teacher surveys were similar but adapted to each respective type of position. Both survey instruments were comprised of seven sections: (1) awareness of DPA policy requirements; (2) perception of DPA policy; (3) scheduling and monitoring of DPA within schools and classrooms; (4) use of DPA resources and supports; (5) perceptions of barriers; (6) self-efficacy; and (7) personal characteristics. Additional questions regarding DPA planning at the school level were also asked of school administrators. School administrators responded on behalf of their school and teachers responded on behalf of their classroom.

#### Outcome variable: Fidelity to DPA policy

The outcome variable for the current analysis was a dichotomous measure of fidelity to the DPA policy at the classroom level based on the required components of the policy [16]. To assess fidelity to the DPA policy at the classroom level, teachers were first asked whether DPA had been implemented in their classroom at least once during the 2013–2014 school year. Respondents indicating “yes” were asked an additional six questions pertaining to the requirements of the DPA policy: 1) duration; 2) frequency; 3) scheduling during instructional time; 4) intensity; 5) continuity; and 6) inclusivity of children with special needs. Responses to the frequency question were measured in number of days (1–5 days) and the other five questions were measured using a five-point Likert scale (never-always). The responses for all six questions were later assigned a value of 1–5: 1 = never/1 day; 2 = rarely/2 days; 3 = sometimes/3 days; 4 = often/4 days; and 5 = always/5 days. The internal consistency reliability of the 6-item initial scale was high (Cronbach’s coefficient alpha = 0.98) [17]. Afterward, an overall score of fidelity was calculated by taking the sum of the participants’ fidelity scores across the six items. The resulting composite score ranged from 0 to 30. A value of zero was assigned when teachers responded that they had not implemented DPA in their classroom at least once during the 2013–2014 school year. The overall fidelity score was later reclassified into “met DPA policy requirements” (scores 24–30) and “did not meet DPA policy requirements” (scores 0 or 6–23.99). Further details regarding this measure can be found in our previous overview paper [17].

#### Independent variables

Independent variables at the school and classroom levels were chosen on the basis of theory, existing literature, and findings from our previous studies [17, 19]. At the school level, five variables were selected from the school administrator survey, while at the classroom-level, eight variables were chosen from the teacher survey. These variables are described, along with their respective coding categories in Table 1.

**Table 1:** Description of independent variables

Variable	Description	Coding Categories
School-level Variables		
School board type	Whether the respondent’s school belongs to a public or Roman Catholic school board	• Public • Roman Catholic
Awareness of DPA policy requirements^a^	The respondent’s awareness of the six components of the DPA policy	• Aware of 4 or more policy requirements • Aware of 3 or less policy requirements
Presence of DPA monitoring procedure	Whether or not the respondent’s school has a procedure for monitoring DPA	• Yes • No
Frequency of using DPA supports^b^	How often the respondent uses available supports (e.g., School Board DPA Committee, public health units) to plan, implement and/or monitor DPA	• Often use/always use • Occasionally use • Never use/rarely use
Competing curriculum priorities^b^	The extent to which the respondent perceives that competing curriculum priorities act as a barrier to implementing DPA at their school	• Disagree/strongly disagree • Neutral • Agree/strongly agree
Classroom-level Variables		
Grade level	Grade level taught by the respondent	• Grade 3 • Grade 5 • Grade 7
Awareness of DPA policy requirements^a^	The respondent’s awareness of the six components of the DPA policy	• Aware of 4 or more policy requirements • Aware of 3 or less policy requirements
Scheduling DPA in teachers’ timetables	Whether or not DPA is scheduled in the respondent’s classroom timetables	• Yes • No
Confidence level in implementing DPA^c^	The respondent’s confidence level in successfully implementing DPA	• High (quite confident/completely confident) • Low-to-moderate (not at all confident/slightly confident/moderately confident)
Frequency of using DPA resources^b^	How often the respondent uses available learning tools (e.g., DPA teacher resource guides, DPA eWorkshop) to help plan, implement and/or monitor DPA	• Often/always use • Occasionally use • Never/rarely use
DPA is realistic and achievable^b^	The extent to which the respondent perceives DPA implementation as being realistic/achievable	• Agree/strongly agree • Neutral • Disagree/strongly disagree
Lack of time^b^	The extent to which the respondent perceives that lack of time acts as a barrier to implementing DPA	• Disagree/strongly disagree • Neutral • Agree/strongly agree
Lack of space^b^	The extent to which the respondent perceives that lack of space acts as a barrier to implementing DPA	• Disagree/strongly disagree • Neutral • Agree/strongly agree

^a^Variable was assessed through a cumulative score of six questions regarding the six components of the DPA policy. The score was dichotomized into two categories for analysis

^b^Responses to the corresponding survey question were measured on a five-point Likert scale, and categorized into a three-level variable for analysis

^c^Responses to the corresponding survey question were measured on a five-point Likert scale, and categorized into a two-level variable for analysis

### Data analysis

As a preliminary step, descriptive analyses were conducted to explore the distribution of responses by school administrators and teachers. Two-level logistic regression models, with random effect on schools, were then carried out to account for the nesting of classrooms within schools. The analysis examined the relative contribution of classroom/teacher characteristics (level 1) and school/administrator characteristics (level 2) on implementation fidelity to DPA policy at the classroom level. Multicollinearity was examined separately for school and classroom predictors using Variance Inflation Factor (VIF) and none of the predictors showed signs of strong multicollinearity (VIF < 10). At first, an unconditional model (i.e., model without any predictors) was fit to calculate the intraclass correlation coefficient (ICC), which shows the amount of variation in the outcome that is attributable to the group level [44]. ICC was calculated using the formula ICC=V_a_/(V_a_ + 3.29)*100, where V_a_ is the area level variance and 3.29 is the estimated individual level variance [44]. The ICC was then used to calculate the design effect. The design effect is the ratio of actual variance for a given design to the variance computed under simple random sampling [45] and it is approximately equal to 1 + (average cluster size - 1) x ICC [46].

Series of models were conducted before arriving at a parsimonious model for implementation fidelity to DPA policy. Initially, a saturated model (Model 1) was fit using all of the school-level and classroom-level predictors. Then independent models were fit with solely school-level predictors (Model 2) and classroom-level (Model 3) predictors. Several other models were carried out by removing predictors that were conceptually less relevant and showed lower effect size compared to other predictors before arriving at the final model (Model 4). The contribution of each model was assessed by comparing the difference in deviance (− 2*log-likelihood) [47] and other model fit statistics. Since deviance tests can only be conducted with nested models, all models presented in this study were compared to the saturated model. Akaike Information Criterion (AIC) and Bayesian Information Criterion (BIC) were used to assess the goodness-of-fit of all four models, including non-nested models. AIC and BIC are both based on the maximum likelihood estimates and control for over-fitting in complex models by penalizing free parameters. Smaller values for both information criteria indicate a better fit model. Statistical significance for all models was determined using alpha level of 0.05. All analyses were carried out in SAS 9.3.

## Results

A total of 209 school administrators and 307 classroom teachers were included in the original study. The current analyses excluded 39 of the school administrators due to lack of participation at the teacher level in corresponding schools. Thus, the analytic sample for the analyses here included 170 school administrators and 307 classroom teachers from the corresponding schools. The number of participating teachers per school ranged from 1 to 3 (mean = 1.8; SD = 0.7).

The distribution of characteristics for the school boards and schools were similar to that of publicly funded elementary schools in Ontario. The majority of school boards for schools included in the study were English (94%) and publicly funded (68%) (Table 2). Approximately three-fourths of the schools were located in urban areas and about half of the schools had 296 students or more enrolled.

**Table 2:** School and administrator characteristics (*n* = 170)

Characteristics	n	%^a^
School Characteristics		
School board Language		
English	159	94
French	11	6
School board Type		
Public	116	68
Roman Catholic	54	32
School location (based on postal code)		
Urban	122	72
Rural	48	28
School size		
Small (≤295 students)	89	52
Large (≥296 students)	81	48
Administrator Personal Characteristics		
Gender		
Male	47	31
Female	103	69
Position		
Principal	152	91
Vice-Principal	15	9
Year of experience as an administrator		
5 years or less	45	30
6 to 15 years	85	57
16 years or more	20	13
Level of health and physical education training		
University-level training	17	11
Little to no training	116	77
Other training	17	11
Priority level of PA in daily life		
High priority	89	59
Moderate priority	48	32
Low priority	14	9
Administrator Responses Regarding DPA		
Awareness of DPA policy requirements		
Aware of 4 or more	136	83
Aware of 3 or less	27	17
Presence of DPA monitoring procedure		
Yes	46	28
No	119	72
Frequency of using DPA supports		
Often/always use	15	9
Occasionally use	63	37
Never/rarely use	91	54
Competing curriculum priorities		
Agree/strongly agree	124	77
Neutral	16	10
Disagree/strongly disagree	22	14

^a^Percentages may not total 100 due to rounding

The majority of administrators were females (69%), principals (91%), and had 6–15 years of experience (57%). Many administrators (59%) viewed PA as a high priority in their daily life, though 77% had little or no health and physical education training. Eighty-three percent of administrators reported awareness of more than half of the DPA policy components. Only a small percentage of administrators reported the presence of a DPA monitoring procedure in their schools (28%) and frequent use of DPA supports (9%). The majority of administrators (77%) perceived competing curriculum priorities as a barrier for DPA implementation.

The demographics and experience levels of teachers were also similar to that of the administrators. The majority of teachers were females (72%) and had 6–15 years of experience (50%). Details of the teachers’ personal characteristics have been published in our previous paper [17]. Table 3 shows teacher-level predictors, including awareness, perceptions, and use of resources related to DPA, by fidelity to DPA policy. As expected, teachers who reported greater confidence level (self-efficacy) in implementation, increased awareness of DPA policy requirements, and frequent use of DPA resources showed higher percentages for meeting DPA policy requirements compared to their counterparts. Three-fourths of the teachers who perceived DPA policy as realistic and achievable met DPA policy requirements in their classrooms. Time and space were amongst the top implementation barriers reported by teachers (data not shown) [17]. Teachers who strongly agreed/agreed that lack of time and lack of space are implementation barriers reported lower percentage of fidelity to DPA policy compared to those who strongly disagreed/disagreed.

**Table 3:** Teacher-level predictors, by fidelity to DPA policy requirements (*n* = 307)

Teacher-Level Predictors	Met DPA Policy Requirements		Did not meet DPA Policy Requirements	
	n^b^	%	n^b^	%
Grade level				
Grade 3	43	43	56	57
Grade 5	56	58	40	42
Grade 7	35	50	35	50
Awareness of DPA policy requirements				
Aware of 4 or more	103	54^b^	86	46
Aware of 3 or less	48	42^b^	65	58
Scheduling DPA in teachers’ timetables				
DPA is scheduled	121	60^b^	82	40
DPA is not scheduled	30	30^b^	69	70
Confidence level in implementing DPA				
High	112	70^b^	49	30
Low-to-moderate	27	25^b^	80	75
Frequency of using DPA resources				
Often/always	25	78^b^	7	22
Occasionally	56	57	42	43
Never/rarely	69	40^b^	103	60
DPA is realistic and achievable				
Agree/strongly agree	97	75^b^	32	25
Neutral	21	44	27	56
Disagree/strongly disagree	32	26^b^	91	74
Lack of time				
Agree/strongly agree	95	41^b^	134	59
Neutral	12	63	7	37
Disagree/strongly disagree	36	84^a^	7	16
Lack of space				
Agree/strongly agree	74	40^a^	109	60
Neutral	20	54	17	46
Disagree/strongly disagree	47	68^a^	22	32

^a^Significant difference in fidelity to DPA requirements at alpha = 0.05

^b^Due to missing values, count totals (n) may not equal total sample (*n* = 307)

Multi-level models showed significant effect by teacher-level predictors on implementation fidelity compared to administrator-level predictors. The ICC value calculated from the unconditional model showed that administrator-level predictors accounted for 5.4% of the variance in implementation of DPA in classrooms. The design effect was 1.04 (1 + (1.8–1)*0.054). MLA is generally not warranted when the design effect is less than two [46]. However, MLA was still carried out in this study due to small cluster sizes and our interest in examining the effects of level-2 variables on fidelity to DPA policy [48].

Findings from the multi-level logistic regression models are shown in Table 4. The saturated model (model 1), with all identified variables, showed teachers’ perception of DPA as realistic and achievable (AOR = 7.40; 95% CI: 2.52–21.76) and teachers’ high confidence in implementing DPA (AOR = 3.08; 95% CI = 1.22–7.76) as the only significant predictors for implementation fidelity to DPA policy. The model with administrator-level predictors alone (model 2) showed no significant findings while the model with only teacher-level predictors (model 3) showed the same significant predictors as the saturated model. In the final model (model 4), three additional variables emerged as significant predictors for implementation fidelity to DPA policy. Teachers’ perception of DPA as realistic and achievable showed the highest odds for implementation. Teachers who strongly agreed/agreed that DPA is realistic and achievable reported 4.63 (95% CI: 1.88–11.44) higher odds for implementing DPA in their classrooms compared to those who strongly disagreed/disagreed. Teachers who reported high confidence level (self-efficacy) in implementing DPA showed 3.39 (95%: 1.47–7.85) higher odds for implementing DPA compared to teachers with low-to-moderate confidence level. Teachers who scheduled DPA in their timetables also reported higher odds (AOR = 2.51; 95% CI: 1.03–6.12) for DPA implementation compared to their counterparts. Those who strongly agreed/agreed that lack of space is a barrier for DPA implementation were 65% less likely to implement DPA policy requirements compared to those who strongly disagreed/disagreed. Teachers who reported a neutral response to lack of time as a barrier were 86% less likely to implement DPA compared to those who disagreed/strongly disagreed.

**Table 4:** Multi-level model for school-level and classroom-level predictors of implementation fidelity to DPA policy

	Model 1	Model 2	Model 3	Model 4
Indicators	AOR (95% CI)	AOR (95% CI)	AOR (95% CI)	AOR (95% CI)
School/Administrator-Level Characteristics				
School board type				
Public	1.25 (0.47–3.29)	1.34 (0.76–2.36)		
Roman Catholic	ref	ref		
Awareness of DPA policy requirements				
Aware of 4 or more	0.61 (0.19–1.97)	0.95 (0.45–2.01)		
Aware of 3 or less	ref	ref		
Presence of DPA monitoring procedure				
Yes	1.46 (0.55–3.86)	1.30 (0.71–2.38)		
No	ref	ref		
Frequency of using DPA supports				
Occasionally	0.90 (0.38–2.16)	1.29 (0.74–2.24)		
Often or always	0.22 (0.04–1.26)	0.70 (0.27–1.86)		
Never or rarely	ref	ref		
Competing curriculum priorities				
Agree/strongly agree	0.85 (0.24–3.00)	0.59 (0.28–1.24)		0.96 (0.34–2.76)
Neutral	0.41 (0.07–2.44)	0.61 (0.20–1.87)		0.48 (0.10–2.25)
Disagree/strongly disagree	ref	ref		ref
Teacher-Level Characteristics				
Grade level				
Grade 3	0.48 (0.17–1.35)		0.51 (0.21–1.27)	0.50 (0.20–1.24)
Grade 5	1.13 (0.40–3.20)		1.46 (0.58–3.64)	1.36 (0.55–3.37)
Grade 7	ref		Ref	ref
Awareness of DPA policy requirements				
Aware of 4 or more	1.24 (0.52–2.95)		1.15 (0.54–2.46)	1.03 (0.47–2.23)
Aware of 3 or less	ref		Ref	ref
Scheduling DPA in teachers’ timetables				
DPA is scheduled	2.11 (0.79–5.64)		2.04 (0.87–4.80)	2.51* (1.03–6.12)
DPA is not scheduled	ref		Ref	ref
Confidence level in implementing DPA				
High	3.08* (1.22–7.76)		3.67* (1.58–8.52)	3.39* (1.47–7.85)
Low-to-moderate	ref		Ref	ref
Frequency of using DPA resources				
Often or always	3.29 (0.54–19.95)		2.20 (0.55–8.75)	
Occasionally	0.94 (0.40–2.19)		0.89 (0.41–1.95)	
Never or rarely	ref		Ref	
DPA is realistic and achievable				
Agree/strongly agree	7.40* (2.52–21.76)		4.65* (1.89–11.43)	4.63* (1.88–11.44)
Neutral	1.61 (0.54–4.75)		1.19 (0.44–3.20)	1.19 (0.44–3.22)
Disagree/strongly disagree	ref		Ref	ref
Lack of time				
Agree/strongly agree	0.25 (0.05–1.24)		0.26 (0.07–1.06)	0.25 (0.06–1.03)
Neutral	0.15 (0.02–1.27)		0.18 (0.03–1.14)	0.14* (0.02–0.84)
Disagree/strongly disagree	ref		Ref	ref
Lack of space				
Agree/strongly agree	0.39 (0.13–1.15)		0.38 (0.14–1.04)	0.35* (0.13–0.97)
Neutral	0.62 (0.15–2.53)		0.53 (0.14–1.95)	0.61 (0.16–2.30)
Disagree/strongly disagree	ref		Ref	ref
Model Fit Statistics				
-2 Log Likelihood	193.3	371.9	229.9	220.7
AIC	237.3	389.9	259.9	250.7
BIC	299.3	417.0	304.2	294.4

*AOR* Adjusted Odds Ratio, *ref*. reference category; * = *p*-value< 0.05

Deviance tests showed model 4 as a better fitting model compared to the saturated model (*p*-value< 0.01). When comparing the fit of all four models, there was disagreement between AIC and BIC. AIC value was lowest for model 1 and BIC value was lowest for model 4. Given that BIC penalizes complex models more than AIC, [49] model 4 was chosen as the more parsimonious model.

## Discussion

The underlying study was the first provincial-level assessment of the status of DPA policy implementation in Canada [17]. The study benefited from access to representative samples of schools and classrooms in Ontario, outcome measures designed to assess fidelity on the six components of the DPA policy, and (for the current analyses) merged data from the two surveys.

A central purpose of the MLA used here was to examine the influences of both school (as reported by administrators) and classroom (as reported by teachers) factors predicting policy fidelity at the classroom level. This is an important consideration since it could be argued that offering DPA (or not) depends largely on decisions of classroom teachers who are the individuals most directly involved in planning and implementing this activity. Yet it is also theoretically and empirically plausible that higher-level (school and administrator) factors could predict classroom fidelity. While a larger number of factors were identified in our earlier examination of predictors of DPA policy implementation in Ontario schools and classrooms, [17] results from MLA suggest that a more limited number of factors predicted implementation in classrooms. Moreover, these are all classroom/teacher-level factors: perception that DPA is realistic and achievable; confidence (self-efficacy) in implementing DPA; scheduling DPA in timetables; lack of space (inversely related) and; lack of time (neutral). None of the school/administrator-level factors that were shown to be significant predictors of school-level policy fidelity in earlier bi-variate analyses proved to be significant predictors of classroom-level policy implementation fidelity when taking both levels of factors into account.

Given these findings, it is tempting to conclude that the significant classroom/teacher- level predictors of classroom policy fidelity are simply reflecting their proximal relationship to the decision to plan and implement DPA in classrooms. To some extent, this may be true. Yet, whether or not the policy is considered to be realistic and achievable may also reflect important (though unmeasured) issues around the structure of schools and schooling: the multiple curricular demands on teachers; emphasis on academic success; and other school-based health and social priorities such as mental health, bullying and safety – all of which may compete to some extent with expectations to offer DPA. Similarly, scheduling DPA on a daily basis indicates attempts to formalize expectations for this activity among a challenging array of externally mandated curriculum expectations and other demands (field trips, assemblies, special events). Lack of space is also clearly situated in objective circumstances and constraints on the extent to which DPA can be accomplished in classrooms containing many desks, chairs and various ages (sizes) of students.

This issue is important on a theoretical level since the results may otherwise appear to indicate that teacher factors alone are the key determinant of policy implementation fidelity. The act of implementing DPA (or not) may be rooted in organizational or structural factors, as suggested by our adapted theoretical framework [17, 21].

Several of the specific factors emerging as significant predictors of DPA in the current study have been identified in other studies of DPA implementation or related initiatives, such as physical education. For example, teacher self-efficacy has been shown to be predictive of implementation of a school-based PA initiative in a British Columbia (Canada) MLA study [25]. As mentioned earlier in the current paper, self-efficacy has also been shown to be associated with PA implementation in a number of descriptive studies and reviews [27, 37, 50]. Studies indicating that availability of time in the curriculum is related to implementation attest to the importance of scheduling DPA and similar initiatives [27, 29, 32, 37]. Also a number of earlier studies found space/facilities to be related to implementation of school PA initiatives [26, 27, 33, 34, 37].

### Limitations

Data from the study were based on self-reports of school administrators and classroom teachers, those most directly responsible for offering DPA in these settings. Their assessments of DPA implementation fidelity were based on responses to questions on each of the component requirements of the policy, thus providing a degree of content validity. However, the data were not validated through direct observation or other means.

Since DPA policies are largely unique to provincial settings in Canada, generalization of the findings to other jurisdictions and initiatives beyond Canada is limited. However, the use of activity breaks in some schools in the US and other countries provides some basis of comparison, and some of the challenges of these are similar to those of DPA. Application of the findings to physical education class is more difficult, since these are usually more highly structured components of the curriculum and more likely to be led by specialist rather than generalist teachers. Even so, there are well-documented reports of the challenges of implementing physical education on a regular basis due to such organizational factors as exemptions and other issues [51, 52].

The present study does not include measures that assess structural factors and influences representing the broader context of these issues. However, we know from our earlier assessment of the development and implementation of DPA in Ontario that there were several political, economic and logistical factors influencing how this initiative was planned and rolled out in Ontario school boards [19]. A key factor was negotiation around providing school boards and schools with sufficient flexibility as to how DPA is delivered, while also adhering to the required components of the policy. Also, while DPA is a provincial policy and curriculum requirement, it “competes” with a number of other priorities and requirements at least partly related to the nature of the education system itself. Understanding these contextual factors helps to explain the current findings – especially with respect to such predictors of fidelity as teachers’ perception about whether or not DPA is realistic and achievable, to what extent it is regularly scheduled in teachers’ timetables, and the availability of adequate space to successfully enact DPA sessions.

While the MLA analyses provide new insights as to the salient factors predicting classroom level DPA, we have not examined here the potential mediating and moderating effects of factors within the model. In order to conduct that analysis, structural equation modelling would be required. Future analysis may address these further relationships. In addition, not yet reported qualitative data from open-ended question items in the teacher survey provide additional insights from teachers as to their practical experiences and constraints in planning and implementing DPA in their classrooms.

## Conclusions

This study provides important contributions to theory and research, with high relevance to application in school health policy and programs in Canada and elsewhere. Since the MLA identified a more parsimonious model of factors predictive of DPA policy fidelity, a clearer picture emerges of the implications for promoting DPA within the education system. Clearly, teacher self-efficacy is a key positive factor, theoretically grounded, and amenable to modification through training, resources and support. Teachers with higher levels of self-efficacy in implementing DPA are more likely to implement it in fidelity with the policy requirements.

Similarly, findings from the MLA indicate that teacher scheduling of DPA is a key predictor of fidelity. Additional support (and monitoring) by school administrators, school boards and the provincial EDU are likely to increase the practice of regular scheduling of DPA in weekly, monthly and annual timetables. Finally, the provision of sufficient space for DPA presents logistical challenges, indicating the need for innovative and creative approaches by administrators, teachers, and others to address this issue. Our findings suggest that these potential changes, accompanied by teacher perspectives that the policy is realistic and achievable, may be related to higher levels of implementation fidelity to the DPA policy in elementary school classrooms. Since several of these factors are imbedded in complex structural and organizational contexts, a systems approach to support these efforts is recommended.

## Abbreviations

AIC: Akaike Information Criterion
BIC: Bayesian Information Criterion
CI: Confidence interval
DPA: Daily Physical Activity (policy)
EDU: Ontario Ministry of Education
ICC: Intraclass correlation coefficient
MLA: Multi-level analysis
MVPA: Moderate to vigorous physical activity
OR: Odds ratio
PA: Physical activity
PPM: Policy/Program Memorandum

## References

[1] Janssen I, Leblanc AG (2010). Systematic review of the health benefits of physical activity and fitness in school-aged children and youth. Int J Behav Nutr Phys Act.

[2] Centers for Disease Control and Prevention (2010). The association between school based physical activity, including physical education, and academic performance.

[3] Biddle SJ, Asare M (2011). Physical activity and mental health in children and adolescents: a review of reviews. BJSM Online.

[4] Institute of Medicine (2013). Educating the student body: taking physical activity and physical education to school.

[5] Rasberry CN, Lee SM, Robin L, Laris BA, Russell LA, Coyle KK (2011). The association between school-based physical activity, including physical education, and academic performance: a systematic review of the literature. Prev Med.

[6] Fedewa AL, Ahn S (2011). The effects of physical activity and physical fitness on children's achievement and cognitive outcomes: a meta-analysis. Res Q Exerc Sport.

[7] Watson A, Timperio A, Brown H, Best K, Hesketh KD (2017). Effect of classroom-based physical activity interventions on academic and physical activity outcomes: a systematic review and meta-analysis. Int J Behav Nutr Phys Act.

[8] World Health Organization (2010). Global recommendations on physical activity for health.

[9] Canadian Society for Exercise Physiology. Canadian 24-hour movement guidelines for children and youth (ages 5-17 years): An integration of physical activity, sedentary behaviour and sleep. http://www.csepguidelines.ca/children-and-youth-5-17. Accessed 21 June 2018.

[10] Allison KR, Adlaf EM, Dwyer JJ, Lysy DC, Irving HM (2007). The decline in physical activity among adolescent students: a cross-national comparison. Can J Public Health.

[11] Hobin E, Erickson T, Comte M, Zuo F, Pasha S, Murnaghan D (2017). Examining the impact of a province-wide physical education policy on secondary students’ physical activity as a natural experiment. Int J Behav Nutr Phys Act.

[12] Barnett TA, O'Loughlin J, Gauvin L, Paradis G, Hanley J (2006). Opportunities for student physical activity in elementary schools: a cross-sectional survey of frequency and correlates. Health Educ Behav.

[13] Lagarde F, LeBlanc C (2010). Policy options to support physical activity in schools. Can J Public Health.

[14] Bassett DR, Fitzhugh EC, Heath GW, Erwin PC, Frederick GM, Wolff DL (2013). Estimated energy expenditures for school-based policies and active living. Am J Prev Med.

[15] Olstad DL, Campbell EJ, Raine KD, Nykiforuk CIJ. A multiple case history and systematic review of adoption, diffusion, implementation and impact of provincial daily physical activity policies in Canadian schools. BMC Public Health. 2015;15(385). 10.1186/s12889-015-1669-6. 10.1186/s12889-015-1669-6 PMC4436021 25885026

[16] Ontario (2005). Ministry of Education: Policy/program memorandum no. 138: Daily physical activity in elementary schools, grades 1–8.

[17] Allison KR, Vu-Nguyen K, Ng B, Schoueri-Mychasiw N, Dwyer JJ, Manson H (2016). Evaluation of daily physical activity (DPA) policy implementation in Ontario: surveys of elementary school administrators and teachers. BMC Public Health.

[18] Cancer Care Ontario, Ontario Agency for Health Protection and Promotion (2012). Taking action to prevent chronic disease: recommendations for a healthier Ontario.

[19] Allison KR, Schoueri-Mychasiw N, Robertson J, Hobin E, Dwyer J, Manson H (2014). Development and implementation of the daily physical activity policy in Ontario, Canada: a retrospective analysis. PHEnex J.

[20] Brownson RC, Colditz GA, Proctor EK (2012). Dissemination and implementation research in health: translating science to practice.

[21] Chaudoir SR, Dugan AG, Barr CHI (2013). Measuring factors affecting implementation of health innovations: a systematic review of structural, organizational, provider, patient, and innovation level measures. Implement Sci.

[22] Durlak JA, DuPre EP (2008). Implementation matters: a review of research on the influence of implementation on program outcomes and the factors affecting implementation. Am J Community Psychol.

[23] Proctor E, Silmere H, Raghavan R, Hovmand P, Aarons G, Bunger A (2011). Outcomes for implementation research: conceptual distinctions, measurement challenges, and research agenda. Adm Policy Ment Hlth.

[24] McLeroy KR, Bibeau D, Steckler A, Glanz K (1988). An ecological perspective on health promotion programs. Health Educ Q.

[25] Mâsse LC, McKay H, Valente M, Brant R, Naylor PJ (2012). Physical activity implementation in schools: a 4-year follow-up. Am J Prev Med.

[26] Brown KMES (2015). ‘It’s not as easy as just saying 20 minutes a day’: exploring teacher and principal experiences implementing a provincial physical activity policy. Univers J Public Health.

[27] Mâsse LC, Naiman D, Naylor PJ (2013). From policy to practice: implementation of physical activity and food policies in schools. Int J Behav Nutr Phys Act.

[28] Robinson DB, Melnychuk NE (2006). A call for PE consultants and specialists: let's get serious about implementing quality PE. PHE J.

[29] Huberty J, Dinkel D, Coleman J, Beighle A, Apenteng B (2012). The role of schools in children’s physical activity participation: staff perceptions. Health Educ Res.

[30] Cardon GM, Van Acker R, Seghers J, De Martelaer K, Haerens LL, De Bourdeaudhuij IMM (2012). Physical activity promotion in schools: which strategies do schools (not) implement and which socioecological factors are associated with implementation?. Health Educ Res.

[31] Leatherdale ST, Manske S, Faulkner G, Arbour K, Bredin C (2010). A multi-level examination of school programs, policies and resources associated with physical activity among elementary school youth in the PLAY-ON study. Int J Behav Nutr Phys Act.

[32] Holt E, Bartee T, Heelan K (2013). Evaluation of a policy to integrate physical activity into the school day. J Phys Act Health.

[33] Gladwin CP, Church J, Plotnikoff RC (2008). Public policy processes and getting physical activity into Alberta's urban schools. Can J Public Health.

[34] Dwyer JJM, Allison KR, LeMoine KN, Faulkner GE, Adlaf EM, Goodman J (2008). A survey of opportunities for school-based physical activity in Ontario elementary schools. PHE J.

[35] Dwyer JJM, Allison KR, Barrera M, Hansen B, Goldenberg E, Boutilier MA (2003). Teachers’ perspective on barriers to implementing physical activity curriculum guidelines for school children in Toronto. Can J Public Health.

[36] Patton I (2012). School-based physical activity in children: an evaluation of the daily physical activity program in Ontario elementary schools.

[37] Naylor PJ, Nettlefold L, Race D, Hoy C, Ashe MC, Wharf Higgins J (2015). Implementation of school based physical activity interventions: a systematic review. Prev Med.

[38] Naiman DI, Leatherdale ST, Gotay C, Masse LC (2015). School factors associated with the provision of physical education and levels of physical activity among elementary school students in Ontario. Can J Public Health.

[39] Shah S, Allison KR, Schoueri-Mychasiw N, Pach B, Manson H, Vu-Nguyen K (2017). A review of implementation outcome measures of school-based physical activity interventions. J Sch Health.

[40] Ontario. Ministry of Education (2006). Healthy schools resource guide: daily physical activity in schools, guide for school principals.

[41] Ontario. Ministry of Education (2005). Healthy schools resource guide: daily physical activity in schools, grades 4 to 6.

[42] Ontario. Ministry of Education (2005). Healthy schools resource guide: daily physical activity in schools, grades 1 to 3.

[43] Ontario. Ministry of Education (2005). Healthy schools resource guide: daily physical activity in schools, grades 7 and 8.

[44] Merlo J, Chaix B, Ohlsson H, Beckman A, Johnell K, Hjerpe P (2006). A brief conceptual tutorial of multilevel analysis in social epidemiology: using measures of clustering in multilevel logistic regression to investigate contextual phenomena. J Epidemiol Community Health.

[45] Statistics Canada. Survey methods and practices. Statistics Canada, Ottawa, Ontario (Canada), 2010. https://www150.statcan.gc.ca/n1/pub/12-587-x/12-587-x2003001-eng.pdf. Accessed 21 June 2018.

[46] Maas CJ, Hox JJ (2005). Sufficient sample sizes for multilevel modeling. Methodology.

[47] Ene M, Leighton EA, Blue GL, Bell BA. Multilevel models for categorical data using SAS PROC GLIMMIX: The basics. Paper 3430-2015. https://support.sas.com/resources/papers/proceedings15/3430-2015.pdf.

[48] Lai MHC, Kwok O (2015). Examining the rule of thumb of not using multilevel modeling: the “design effect smaller than two” rule. J Exp Educ.

[49] Hastie T, Tibshirani R, Friedman J (2009). Data mining, inference, and prediction. The elements of statistical learning.

[50] Bowins W, Beaudoin C (2011). Experienced physical education teachers adapting to a new curriculum: perceived facilitators and inhibitors. PHEnex J.

[51] Carlson JA, Sallis JF, Chriqui JF, Schneider L, McDermid LC, Agron P (2013). State policies about physical activity minutes in physical education or during school. J Sch Health.

[52] Taber DR, Chriqui JF, Chaloupka FJ (2013). State laws governing school meals and disparities in fruit/vegetable intake. Am J Prev Med.

